# Effect of educational intervention on medication timing in Parkinson's disease: a randomized controlled trial

**DOI:** 10.1186/1471-2377-7-20

**Published:** 2007-07-16

**Authors:** Katherine A Grosset, Donald G Grosset

**Affiliations:** 1 Department of Neurology, Institute of Neurological Sciences, Southern General Hospital, Glasgow, UK

## Abstract

**Background:**

Medicine usage in Parkinson's disease patients is often imperfect, in particular irregular timing of medication. The effect of informing Parkinson's disease patients about the continuous dopaminergic hypothesis (to encourage regular medicine intake) on medication adherence and motor control was tested.

**Methods:**

Patients were randomised either to the active group (receiving the intervention) or control group (no extra information). Antiparkinson medicine usage was monitored for 3 months before and after the intervention using electronic pill bottles which record the date and time of opening (MEMS^®^, Aardex, Switzerland) and data used to calculate the percentage of doses taken at correct time intervals.

**Results:**

43 patients (52%) were randomised to active counselling, and 40 (48%) were controls (standard management). The intervention effect (difference in timing adherence pre- to post-intervention between the 2 groups) was 13.4% (CI 5.1 to 21.7), p = 0.002. Parkinson motor scores did not change significantly (active group 0.1, CI -3.4 to 3.7) versus controls (4.5, CI 1.6 to 7.1), p = 0.06.

**Conclusion:**

Timing adherence, but not motor scores, improves by providing patients with extra information. Therapy timing is of potential importance in Parkinson's disease management.

**Trial registration number:**

NCT00361205

## Background

Patients with Parkinson's disease (PD) depend on medication for relief of motor symptoms, and for this reason are often assumed to medicate very carefully. Overall, medication adherence is very good, but a subset of 15 to 20% of cases take less than 80% of the *total *prescribed dose[1,2], although the limit of 80% of tablet intake is often applied, this is arbitrary and does not have a strong pharmacological basis[3]. However, irregular *timing *of drug ingestion is almost universal[2], perhaps contributed by fluctuating symptoms and drug regimen complexity. Pulsatile dopaminergic stimulation in the basal ganglia is implicated in the development and manifestation of motor complications of advancing PD[4]. The mechanism of motor complications is complex but may relate partly to erratic absorption and short half-life of levodopa causing fluctuating serum and brain drug levels and abnormal pulsatile stimulation of striatal dopamine receptors [5,6] contrasting with more continuous neurone firing under normal circumstances [7,8]. In early disease the dopamine neurones have the capacity to buffer variations in striatal dopamine levels, but as the disease progresses fluctuating plasma dopamine levels correlate with alternating high and low striatal dopamine levels causing pulsatile stimulation clinically manifesting as emerging motor fluctuations [9].

Irregular medication intake is likely to contribute to peaks and troughs in serum and brain drug levels. In other diseases, patient adherence to prescribed medication improves through simplifying drug regimens[10,11], providing additional education[12,13], counselling and behavioural approaches [14-16] and providing reminder packaging[17]. We tested the effect on the timing of medicine ingestion of an educational approach, in which patients were given detailed additional information about the continuous dopaminergic theory[4].

## Methods

Patients attending a regional movement disorder clinic with idiopathic PD (by UK Brain Bank criteria)[18] and prescribed one or more antiparkinson drug (including dopamine agonist or levodopa) were invited to participate. Patients who were unable to manipulate the electronic pill monitoring bottles, or whose adherence would be adversely affected by using the electronic pill monitoring bottles (e.g. those reliant on an adherence aid) were excluded. If a carer normally assisted with patient's medication, they were asked to use the MEMS containers. The study received ethics approval from the South Glasgow Hospitals Ethics Committee and signed consent was obtained. The informed consent procedure was the same for active and control groups, and patients were not advised that some would receive special educational instructions.

Patients were randomly assigned (computer generated and placed in opaque envelopes) to either the active (counselled) or control groups. Randomisation preceded baseline clinical assessment and issuing of MEMS bottles. Baseline assessments of unified Parkinson's disease rating scale (UPDRS)[19], Hoehn and Yahr[20], Schwab and England[21], mini-mental state examination[22], geriatric depression score[23] and quality of life score (PDQ 39)[24] were performed. All clinical recordings were blind to patient group and performed in an 'on' state. The UPDRS 3 and adverse events were recorded at each visit. The quality of life score (PDQ 39) was repeated at the final visit.

All antiparkinson drugs were monitored during two 3 month periods (before and after the educational intervention) using electronic monitoring pill bottles (MEMS^®^, Aardex, Switzerland), that record the time and date of bottle opening. Individual tablet strengths were given a MEMS container e.g. ropinirole 7 mg three times daily required one MEMS container for each of 5 mg and 2 mg tablets.

After the first 3-month period of MEMS monitoring, patients in the active group were given verbal and written information about the continuous dopaminergic theory (one A4 page, Additional file 1), and tailored written guidance on optimal medicine timing for their drug regimen. The counselling (by one investigator, KG) explained that in health, brain dopamine is constant, and that fluctuations from Parkinson's medications should be minimised to simulate normal dopamine levels. At the time of the intervention, individual baseline MEMS results had not been processed, and all patients in the active group were given the same information. Control patients received standard care, but also had medication intake monitored using the MEMS device. All patients were seen every 3 months. During the study medication was adjusted according to clinical need. The increase in levodopa equivalent units during the study period was calculated according to established formula[25].

Timing adherence (the percentage of doses taken at the correct time interval) was calculated using time intervals during a 24-hour day which optimise the pharmacokinetic profile, plus a 25% allowance, eg. 3 times daily medication is satisfactory at between 6 and 10 hours Selegiline 5 mg twice daily was excluded from analysis as the second dose is taken at lunchtime to avoid sleep disturbance. Adherence data were downloaded from the MEMS containers using Powerview^® ^software (Aardex, Switzerland).

### Analysis

Data are presented as mean and 95% confidence interval (CI) when normally distributed, otherwise median and interquartile (IQ) range. Groups were compared using unmatched t-tests for parametric data and Mann-Whitney for non-parametric data. The primary end point was a difference in timing adherence between groups. Analysis was carried out by intent to treat (using last observation carried forward, and patients completing the baseline monitoring). To detect a difference of 15% after intervention, using an estimated SD of 20% for timing compliance, gave a sample size of 58 patients (80% power, two-sided). This 15% difference was arbitrary, since clinical significance is not known in PD. To allow for drop-out, it was planned for 80 patients to be recruited. Secondary end points were differences in changes in UPDRS 3 and quality of life (PDQ 39) between groups. Statistical analysis used Prism 3 (GraphPad^®^, CA, USA).

## Results

**Figure 1:**
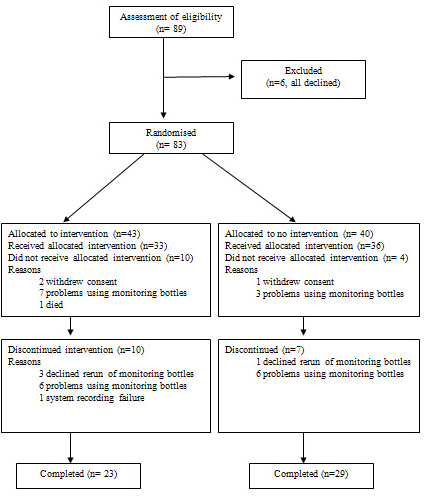
CONSORT diagram of study.

Of 89 patients asked to participate, 6 (7%) declined (Figure 1). Of the remaining 83, 43 were randomised to the active group and 40 to the control group. Fourteen patients dropped out during the first 3-month monitoring period, 10 from the active group (2 withdrew consent after baseline assessment, 1 died, and 7 had problems with the electronic monitoring bottles).

Four control patients dropped out (3 had problems with the bottles, 1 withdrew consent). There were no significant differences in baseline characteristics in patients who dropped out versus those continuing. Baseline adherence data were therefore available for 69 patients (33 in the active group and 36 in the controls, Table 1).

**Table 1:** Patient baseline characteristics by group

	Pre-Intervention		Post-Intervention	
	Active (n = 33)	Control (n = 36)	Active (n = 23)	Control (n = 29)
Males (%)	62%	51%		
Age (years)	61 (10)	66 (13)		
Prescribed levodopa (%)	21 (62%)	25 (71%)	18 (78%)	22 (76%)
Levodopa dose (mg)	485 (252)	538 (389)	511 (306)	670 (380)
Prescribed dopamine agonist (%)	25 (74%)	25 (71%)	18 (78%)	18 (62%)
Change in levodopa equivalent units	-	-	51 (148)	70 (149)
Number of PD drugs	2.4 (1.1)	2.4 (1.3)	2.4 (1.2)	2.2 (1.5)
Number of PD daily doses	4.0 (2.5)	4.0 (1.3)	4 (0.8)	4 (1.2)
Number of PD tablets per day	9 (5)	9.1 (5)	9.5 (5)	9 (5)
Number of non-PD drugs per day	2.6 (3)	2.9 (1.8)	2.5 (3)	3.5 (4)
Total number of tablets per day	12 (5)	12 (5)	13 (7)	12 (7)
Number of patients with carer	6 (18%)	7 (19%)		
Duration of PD (years)	7.5 (6)	6.3 (4.1)	-	-
UPDRS 3	30 (12)	24 (13)	29 (14)	28 (14)
Hoehn & Yahr	2.4 (0.7)	2.4 (0.7)	2.5 (0.7)	2.5 (0.7)
Schwab & England	78 (10)	76 (14)	71 (18)	73 (15)
MMSE	28 (2)	28 (2)	-	-
Geriatric depression score	12 (6)	10 (7)	-	-
PDQ SI	30 (15)	26.5 (18)	36 (15)	28 (14)
Timing adherence, median (IQ)	17% (9–51)	21% (10–59)	39% (22–58)	20% (10–47)*

Data are mean (standard deviation) unless otherwise stated. PD = Parkinson's disease, UPDRS = Unified Parkinson's disease rating scale, MMSE = mini mental state examination, PDQ 39 SI = Parkinson's disease quality of life single index, IQ = interquartile range. *p = 0.007. There were no other significant differences between groups.

**Figure 2:**
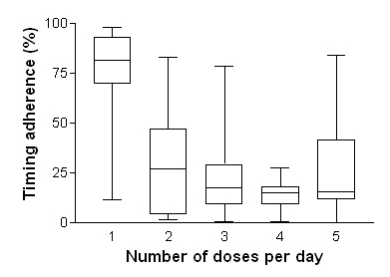
Adherence (maximum, upper quartile, median, lower quartile and minimum) against number of daily doses of antiparkinson medication. Timing adherence was lower with increasing number of daily doses (p < 0.0001). Data are from 69 patients in the pre-intervention phase of the study.

At baseline, timing adherence was a median of 17% (IQ 9–51) in the active group versus 21% (IQ 10–59) for controls, a non-significant difference. Other parameters did not differ significantly between active and control groups (Table 1). Timing adherence was significantly better for once daily drugs (median 82%, IQ 70–93) than drugs prescribed twice daily (33%, IQ 4–47) or more frequently (p < 0.0001)(Figure 2).

In the post-intervention period 17 patients dropped out, 10 from the active group, and 7 from the control group (Figure 1). Post-intervention adherence data were evaluable for 52 patients (23 active, 29 control)(Table 1). After the intervention timing adherence was significantly better in the active group at a median of 39% (IQ 22–58) compared to a median of 20% (IQ 10–47) in the control group (p = 0.007). The intervention effect (difference in timing adherence pre- to post-intervention between the 2 groups) was 13.4% (CI 5.1 to 21.7), p = 0.002. After excluding drugs taken once daily from analysis, the intervention effect was 23.1% (CI 11.7 to 34.5), p = 0.0001. There were no significant differences between UPDRS 3 or quality of life scores or adverse events between groups before intervention (Table 1). The PDQ single index score increased by a mean of 6.0 (CI 2.3 to 9.7) for the active group, versus a mean change of 3.5 (CI -1.6 to 8.6) in controls (p = 0.4). The mean change in UPDRS 3 was 0.1 (CI -3.4 to 3.7) in the active group versus 4.5 (CI 1.6 to 7.1) in controls (p = 0.06).

There were no significant differences in frequency or type of adverse events between groups (active group 1.5 adverse events per patient, versus 1.1 in controls). The commonest adverse effects were in declining frequency: insomnia, sleepiness, dyskinesia, and nausea.

## Discussion

This is the first study to report an improvement in the timing of tablet intake in Parkinson's Disease (through any mechanism), but there was no change in Parkinson motor score, or quality of life scores. The response in timing adherence was however variable, between a 23% worsening and a 96% improvement in actively counselled patients, and control patients showed significantly worse overall medication timing. This may be due in part to waning novelty of the technique, between the first and second 3-month monitoring period. Deterioration in control group adherence figures post-intervention is reported elsewhere[26]. The majority of patients found regularisation of medicine-taking difficult; reasons include forgetfulness and are well documented in PD[1].

Timing adherence is based on a 24 hour day, rather than waking hours. This matches the concepts of the continuous dopaminergic theory, namely that smoother symptom control results from continuous, rather than pulsatile, drug delivery. Although patients frequently medicate preferentially in the daytime, which lowers their timing adherence, we did not adjust our results to account for this. The overnight problems of Parkinson symptoms, and the continuous dopaminergic theory, both argue against such an approach.

Drop-out from studies with electronic pill-bottle monitoring is expected[27]. Although adverse events may contribute, this is commoner following therapy initiation[28], while our patients were on established medication. Our monitoring was prolonged and repeated, typically encompassing several medications taken several times a day, which contrasts with studies of monotherapy or twice-daily medication[29]. Limitations in our study are as follows. Firstly, a substantial proportion of patients were unable or unwilling to use MEMS containers (totalling 27 of 83 patients, 32.5%), which reduces adherence data for intention to treat analysis of the primary outcome. However, clinical scoring was continued in all of these cases. Secondly, the level at which sub-optimal adherence becomes clinically important in PD is not known, and our study does not assist in defining this. Thirdly, we underestimated the variability of timing adherence, which was unreported at the time of planning this study. This should be considered in designing further studies in this area.

Many interventions to improve therapy adherence have been tested[30], but this is the first such study in Parkinson's disease. The vast majority of studies used pill counts, self report or physician/nurse assessment to measure adherence despite well-recognised shortcomings of these methods [31]. Most interventions involve multiple components[30], leaving uncertainty as to which aspects have a positive effect. We therefore chose an intervention with 3 components (verbal and written information, and tailored timing guidance) but had a single focus of improving timing adherence. The resulting timing adherence improvement was similar to that from individualised cue-dose training (linking medicine taking to daily activities) in diabetes[14]. Another interventional approach is to inform the patient of their own prior dosing history from the electronically monitored data[32].

In our study drugs taken once daily were taken more regularly than more complicated regimens, which is consistent with a systematic review of 76 electronic monitoring studies[33]. Pharmaceutical development of more once daily antiparkinson preparations may help ease the process of medicine taking.

There was no significant difference in UPDRS 3 before and after the intervention. Quality of life deteriorated, but is more influenced by non-motor factors such as depression. We did not find an association between deterioration in domains of the PDQ score and UPDRS changes, in particular considering items which might respond to increasing, or regularising, dopaminergic therapy (bodily discomfort, mobility). Clinical improvement is reported in some adherence studies (e.g. epilepsy[34]), but is by no means universal[14].

## Conclusion

Timing adherence improves by providing patients with extra information. Therapy timing is of potential importance in Parkinson's disease management. Further larger, longer-term studies are necessary to determine whether improved medication timing is sustainable, and whether there are beneficial effects associated with the regularity of medication intake in Parkinson's disease.

## Abbreviations

CI = confidence interval, IQ = interquartile range, MEMS = medication event monitoring system (the electronic monitoring bottles), MMSE = mini-mental state examination, PD = Parkinson's disease, PDQ 39 = Parkinson's disease quality of life questionnaire, UPDRS = unified Parkinson's disease rating score.

## Competing interests

The author(s) declare that they have no competing interests.

## Authors' contributions

KG participated in the study design, carried out the co-ordination of the study, participated in data collection, performed the statistical analysis and drafted the manuscript. DG conceived of the study, participated in its design, participated in data collection, participated in the statistical analysis and participated in draft revision of the manuscript. All authors read and approved the final manuscript.

## Pre-publication history

The pre-publication history for this paper can be accessed here:



## Supplementary Material

Additional File 1 Why it is important to take medication for Parkinson's disease on a regular basis. Written information given to patients in active intervention group. Click here for file

## References

[B1] Leopold NA, Polansky M, Hurka MR (2004). Drug adherence in Parkinson's disease. Mov Disord.

[B2] Grosset KA, Bone I, Grosset DG (2005). Suboptimal medication adherence in Parkinson's disease. Mov Disord.

[B3] Hughes DA, Bagust A, Haycox A, Walley T (2001). Accounting for noncompliance in pharmacoeconomic evaluations. Pharmacoeconomics.

[B4] Stocchi F, Vacca L, Ruggieri S, Olanow CW (2005). Intermittent vs continuous levodopa administration in patients with advanced Parkinson disease: a clinical and pharmacokinetic study. Arch Neurol.

[B5] Bezard E, Brotchie JM, Gross CE (2001). Pathophysiology of levodopa-induced dyskinesia: potential for new therapies. Nat Rev Neurosci.

[B6] Jenner P (2000). Pathophysiology and biochemistry of dyskinesia: clues for the development of non-dopaminergic treatments. J Neurol.

[B7] Grace AA (1991). Phasic versus tonic dopamine release and the modulation of dopamine system responsivity: a hypothesis for the etiology of schizophrenia. Neuroscience.

[B8] Onn SP, West AR, Grace AA (2000). Dopamine-mediated regulation of striatal neuronal and network interactions. Trends Neurosci.

[B9] Spencer SE, Wooten GF (1984). Altered pharmacokinetics of L-dopa metabolism in rat striatum deprived of dopaminergic innervation. Neurology.

[B10] Melikian C, White TJ, Vanderplas A, Dezii CM, Chang E (2002). Adherence to oral antidiabetic therapy in a managed care organization: a comparison of monotherapy, combination therapy, and fixed-dose combination therapy. Clin Ther.

[B11] Girvin B, McDermott BJ, Johnston GD (1999). A comparison of enalapril 20 mg once daily versus 10 mg twice daily in terms of blood pressure lowering and patient compliance. J Hypertens.

[B12] Peveler R, George C, Kinmonth AL, Campbell M, Thompson C (1999). Effect of antidepressant drug counselling and information leaflets on adherence to drug treatment in primary care: randomised controlled trial. BMJ.

[B13] Henry A, Batey RG (1999). Enhancing compliance not a prerequisite for effective eradication of Helicobacter pylori: the HelP Study. Am J Gastroenterol.

[B14] Rosen MI, Rigsby MO, Salahi JT, Ryan CE, Cramer JA (2004). Electronic monitoring and counseling to improve medication adherence. Behav Res Ther.

[B15] Weber R, Christen L, Christen S, Tschopp S, Znoj H, Schneider C, Schmitt J, Opravil M, Gunthard HF, Ledergerber B (2004). Effect of individual cognitive behaviour intervention on adherence to antiretroviral therapy: prospective randomized trial. Antivir Ther.

[B16] O'Donnell C, Donohoe G, Sharkey L, Owens N, Migone M, Harries R, Kinsella A, Larkin C, O'Callaghan E (2003). Compliance therapy: a randomised controlled trial in schizophrenia. BMJ.

[B17] Becker LA, Glanz K, Sobel E, Mossey J, Zinn SL, Knott KA (1986). A randomized trial of special packaging of antihypertensive medications. J Fam Pract.

[B18] Gibb WR (1988). Accuracy in the clinical diagnosis of parkinsonian syndromes. Postgrad Med J.

[B19] Fahn S, Elton R, members of the UPDRS Development Committee (1987). Unified Parkinson's disease rating scale. Recent develpoments in Parkinson's disease.

[B20] Hoehn MM, Yahr MD (1967). Parkinsonism: onset, progression and mortality. Neurology.

[B21] Schwab RS, England AC (1961). Parkinson's disease: rehabilitation aspects. Rehabil Lit.

[B22] Folstein M, Folstein S, McHugh P (1975). Mini-mental state. A practical method for grading the cognitive state of patients for the clinician.. J Psychiatr Res.

[B23] Yesavage JA, Brink TL, Rose TL, Lum O, Huang V, Adey M, Leirer VO (1982). Development and validation of a geriatric depression screening scale: a preliminary report. J Psychiatr Res.

[B24] Jenkinson C, Fitzpatrick R, Peto V, Greenhall R, Hyman N (1997). The Parkinson's Disease Questionnaire (PDQ-39): development and validation of a Parkinson's disease summary index score. Age Ageing.

[B25] Parkin SG, Gregory RP, Scott R, Bain P, Silburn P, Hall B, Boyle R, Joint C, Aziz TZ (2002). Unilateral and bilateral pallidotomy for idiopathic Parkinson's disease: a case series of 115 patients. Mov Disord.

[B26] Fulmer TT, Feldman PH, Kim TS, Carty B, Beers M, Molina M, Putnam M (1999). An intervention study to enhance medication compliance in community-dwelling elderly individuals. J Gerontol Nurs.

[B27] Hugen PW, Langebeek N, Burger DM, Zomer B, van Leusen R, Schuurman R, Koopmans PP, Hekster YA (2002). Assessment of adherence to HIV protease inhibitors: comparison and combination of various methods, including MEMS (electronic monitoring), patient and nurse report, and therapeutic drug monitoring. J Acquir Immune Defic Syndr.

[B28] Bull SA, Hunkeler EM, Lee JY, Rowland CR, Williamson TE, Schwab JR, Hurt SW (2002). Discontinuing or switching selective serotonin-reuptake inhibitors. Ann Pharmacother.

[B29] Lee JY, Kusek JW, Greene PG, Bernhard S, Norris K, Smith D, Wilkening B, Wright JT (1996). Assessing medication adherence by pill count and electronic monitoring in the African American Study of Kidney Disease and Hypertension (AASK) Pilot Study. Am J Hypertens.

[B30] Haynes RB, McDonald H, Garg AX, Montague P (2002). Interventions for helping patients to follow prescriptions for medications. Cochrane Database Syst Rev.

[B31] Cramer JA, Mattson RH, Prevey ML, Scheyer RD, Ouellette VL (1989). How often is medication taken as prescribed? A novel assessment technique. JAMA.

[B32] Vrijens B, Belmans A, Matthys K, de Klerk E, Lesaffre E (2006). Effect of intervention through a pharmaceutical care program on patient adherence with prescribed once-daily atorvastatin. Pharmacoepidemiol Drug Saf.

[B33] Claxton AJ, Cramer J, Pierce C (2001). A systematic review of the associations between dose regimens and medication compliance. Clin Ther.

[B34] Cramer JA, Glassman M, Rienzi V (2002). The relationship between poor medication compliance and seizures. Epilepsy Behav.

