# Supplementary material for: Effect of educational intervention on medication timing in Parkinson's disease: a randomized controlled trial
Source: BMC Neurol. 2007 Jul 16;7:20. doi: 10.1186/1471-2377-7-20 (PMC1931606; doi:10.1186/1471-2377-7-20)
Supplement: Additional File 1 — Why it is important to take medication for Parkinson's disease on a regular basis. Written information given to patients in active intervention group. [file 1471-2377-7-20-S1.doc]

WHY IT IS IMPORTANT TO TAKE MEDICATION FOR PARKINSON’S DISEASE ON A REGULAR BASIS

In Parkinson’s disease, there is a loss of brain nerve cells containing a chemical called dopamine. The dopamine is needed to help control movements. Parkinson’s medication either replaces the dopamine, boosts the dopamine by preventing its breakdown, or stimulates the brain in the same way as dopamine. Normally, the brain is stimulated by dopamine in a continuous fashion. In patients with Parkinson’s disease, the dopamine levels are too low. When medication is taken, the levels of dopamine increase and are high after 30-60 minutes, the levels then gradually fall as the body breaks down the dopamine. This causes high and low levels of dopamine in the brain. If tablets are taken at regular intervals, there are smoother levels of dopamine in the brain giving smoother control of symptoms. Taking medicines at regular time intervals may help prevent the development of long term side effects in the future.

Unfortunately, sometimes after taking medicine for Parkinson’s disease for several years, variations or fluctuations occur in the control of the symptoms. When the symptoms are well controlled and you are able to function, this is termed “on”, if the symptoms are poorly controlled and the movements are slow and stiff, this is termed “off”. When the symptoms fluctuate this is called the “on/off” effect. If medications are taken on a regular basis, there is a steadier supply of dopamine to the brain and these fluctuations can be minimised.
